# UTILITY OF SIX-MONTH PROGNOSIS TO PREDICT SURVIVAL AMONG NEWLY ADMITTED NURSING HOME RESIDENTS WITH CANCER

**DOI:** 10.1093/geroni/igac059.2318

**Published:** 2022-12-20

**Authors:** Long Vu, David Warner, Nicholas Schiltz, Johnie Rose, Jennifer Cullen, Cynthia Owusu, Martha Sajatovic, Sara Douglas

**Affiliations:** Case Western Reserve University, Cleveland, Ohio, United States; University of Alabama at Birmingham, Birmingham, Alabama, United States; Case Western Reserve University, Cleveland, Ohio, United States; Case Western Reserve University School of Medicine, Cleveland, Ohio, United States; Case Western Reserve University School of Medicine, Cleveland, Ohio, United States; Case Western Reserve University School of Medicine, Cleveland, Ohio, United States; Case Western Reserve University School of Medicine, Cleveland, Ohio, United States; Case Western Reserve University, Cleveland, Ohio, United States

## Abstract

A prognosis of less than 6 months life expectancy has potential to be useful in planning and evaluating end-of-life care; however, its agreement with actual survival data has not been assessed. Here, we measured the sensitivity, specificity, positive predictive value (PPV), and negative predictive value (NPV) of the nursing home (NH) Minimum Data Set (MDS) 6-month prognosis indicator among individuals diagnosed with incident breast (female), colorectal, lung, pancreatic, or prostate cancer during the years 2004-2017 and admitted to a NH between 2012-2017 (n=254,183). Cancer patients were identified from the linked Surveillance, Epidemiology, and End Results (SEER) Registry. The median age was 80 years, 52.6% were women, and 15.15% were non-White. Only 7,541 (2.97%) of patients were deemed by a physician to have a life expectancy of less than 6 months as recorded in their first comprehensive MDS assessment; of those, 83.0% died within 6 months, and 12.3% died after 6 months. Sensitivity and specificity were 7.8% and 99.3%, respectively, for the prognosis indicator predicting mortality within 6 months; PPV was 83.0% and NPV was 69.9%. In conclusion, although very few patients had received a physician designation of their 6-month prognosis, a high percentage actually died within 6 months. Conversely, among those without a 6-month prognosis, nearly one-third died within 6 months. A terminal diagnosis appears reasonably predictive of death — when made. However, its low sensitivity demonstrates that a vast majority of terminal patients are not properly captured, calling into question its greater application.

